# Supplementary figures and images for: Reprogramming the EnvZ-OmpR two-component system confers ethanol tolerance in Escherichia coli by stabilizing the outer membrane and altering ferric homeostasis
Source: PLoS Genet. 2025 Dec 17;21(12):e1011707. doi: 10.1371/journal.pgen.1011707 (PMC12742742; doi:10.1371/journal.pgen.1011707)

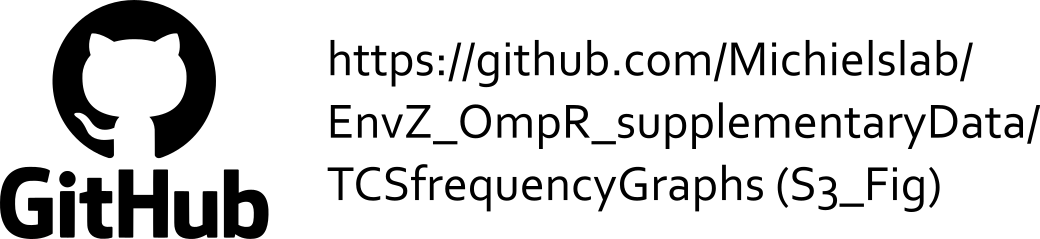

Supplement: S3 Fig — The reader can find all individual graphs at https://github.com/Michielslab/EnvZ_OmpR_supplementaryData as svg files. (TIFF) [file pgen.1011707.s003.tiff]

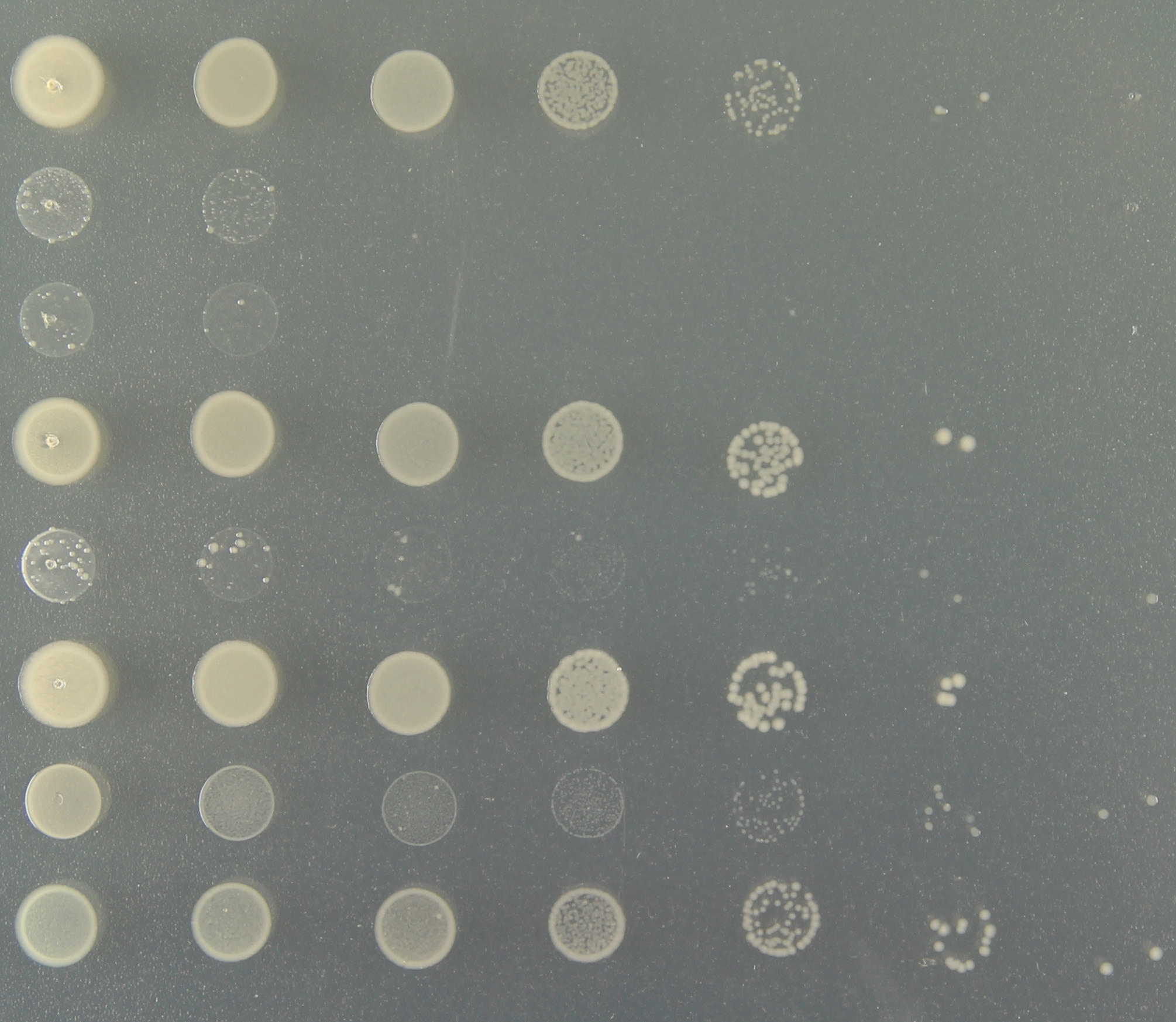

Supplement: S11 Fig — (TIF) [file pgen.1011707.s011.tif]

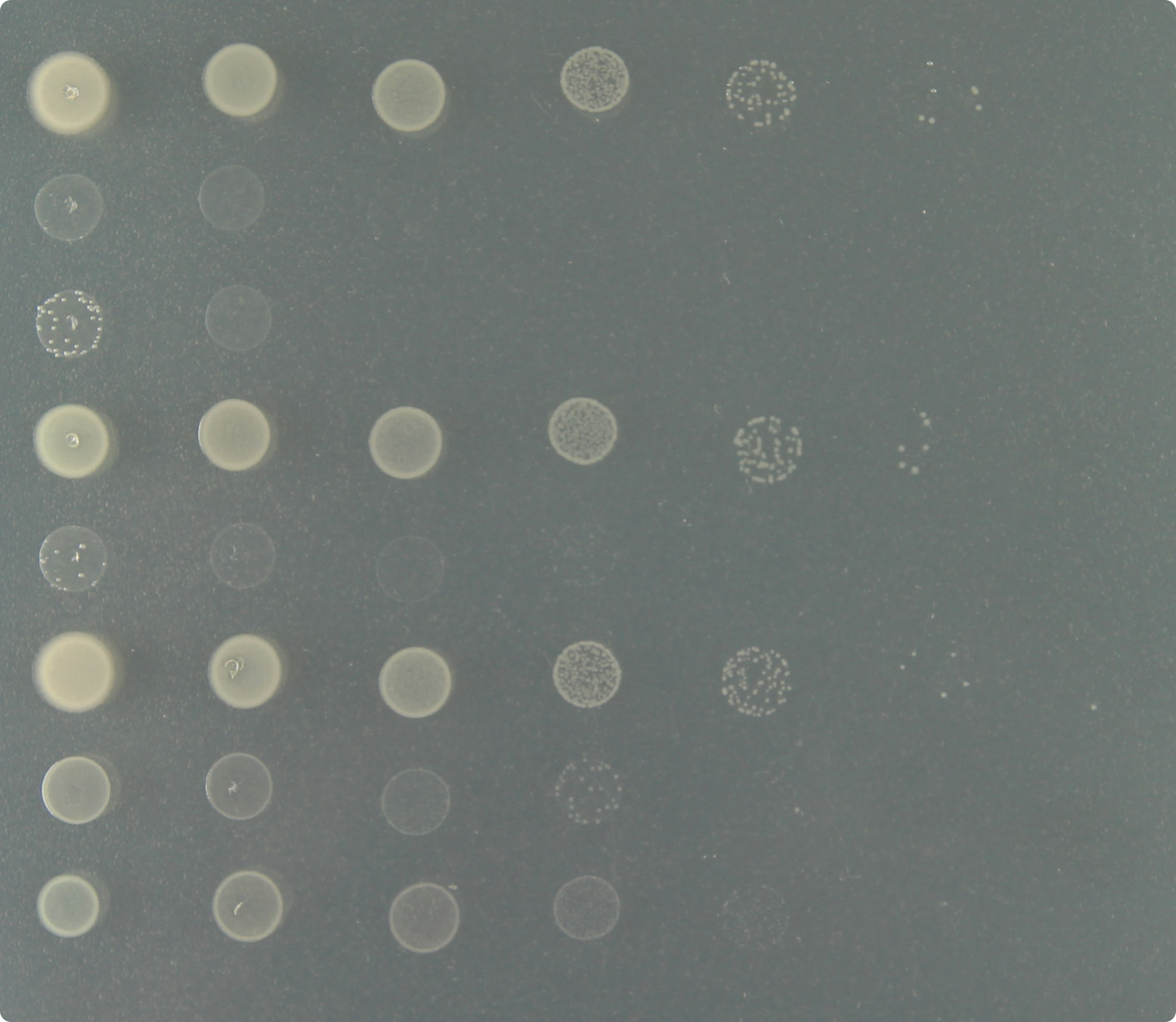

Supplement: S12 Fig — (TIF) [file pgen.1011707.s012.tif]

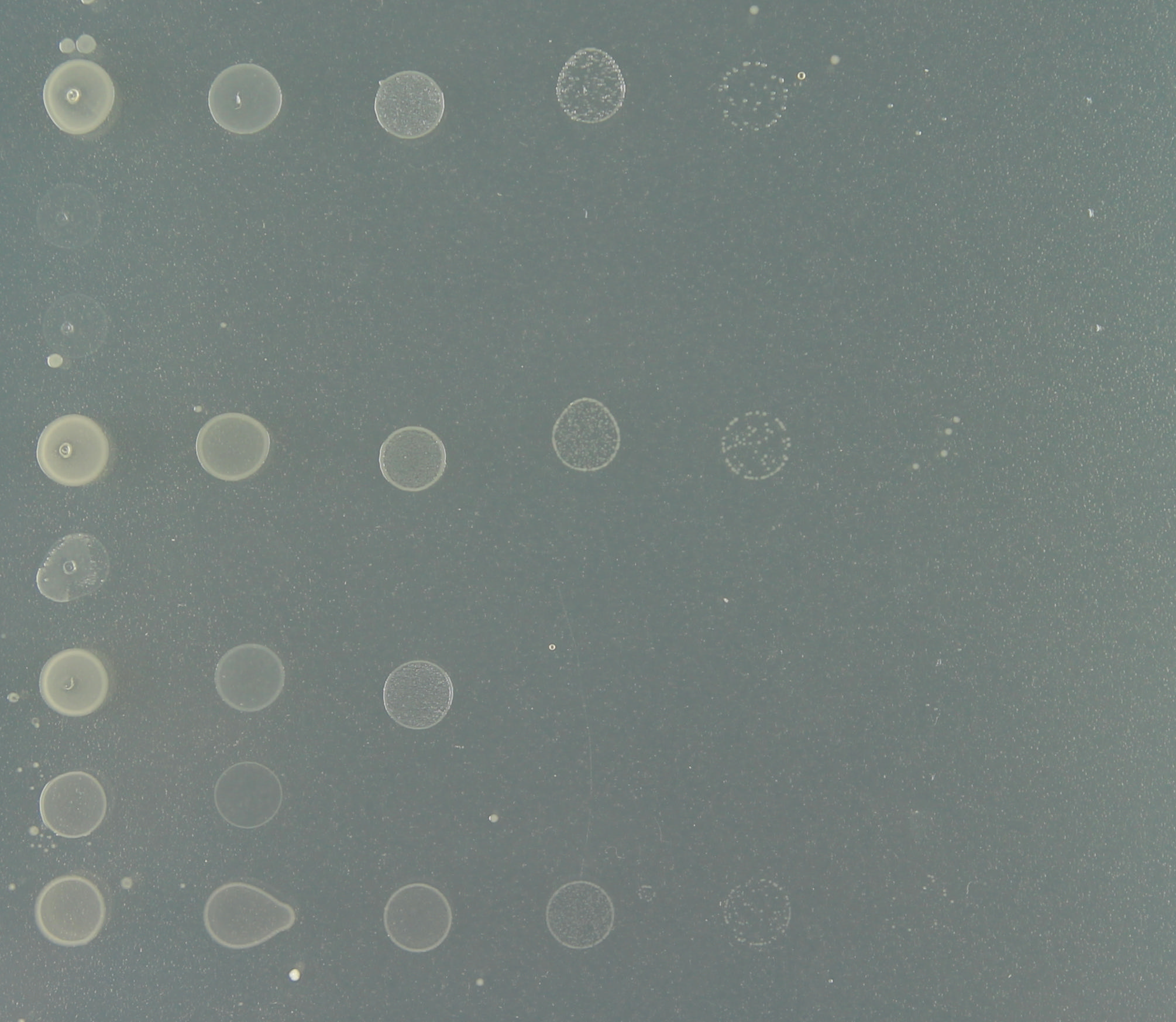

Supplement: S13 Fig — (TIF) [file pgen.1011707.s013.tif]

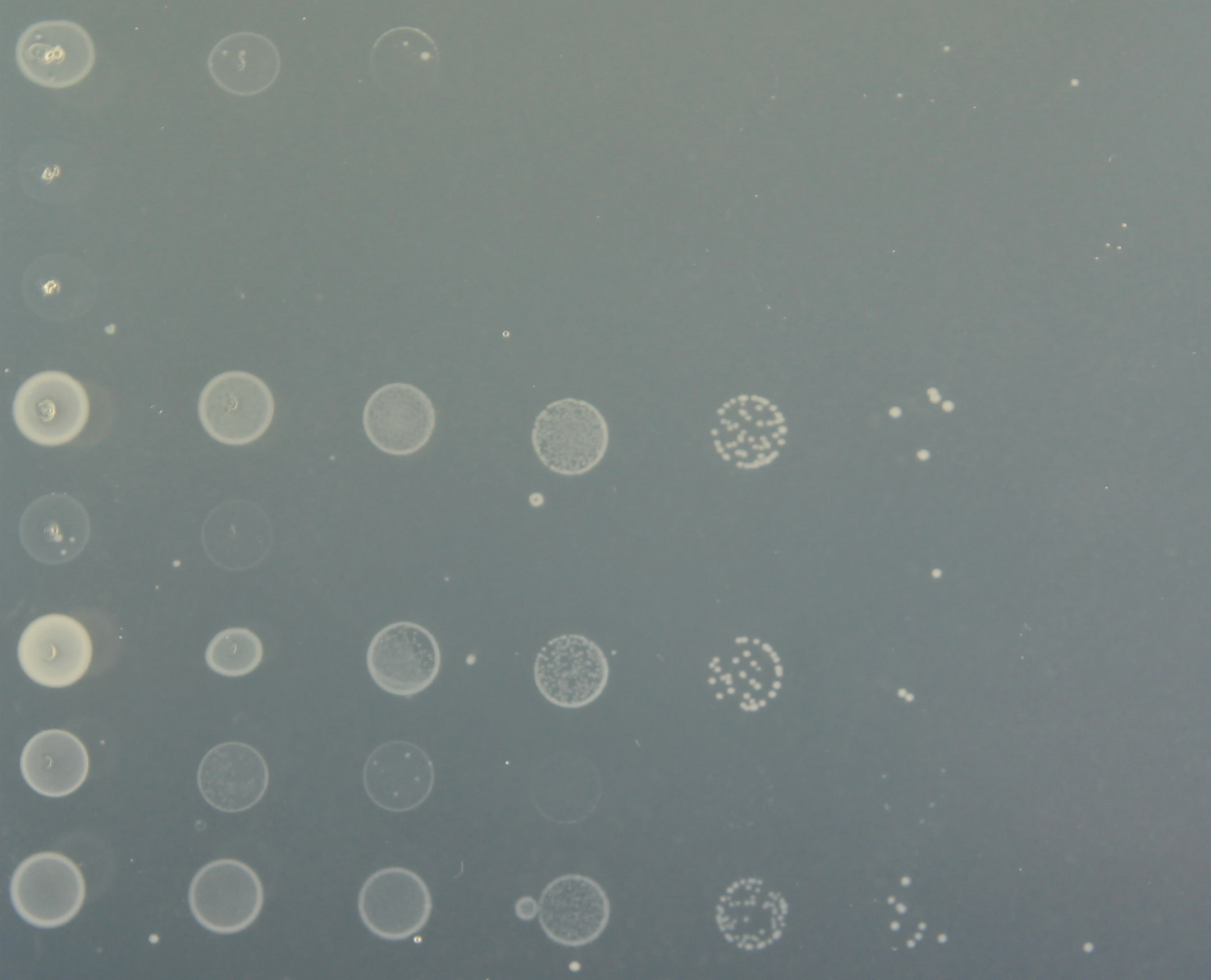

Supplement: S14 Fig — (TIF) [file pgen.1011707.s014.tif]
